# Intimate partner abuse before and during pregnancy as risk factors for postpartum mental health problems

**DOI:** 10.1186/1471-2393-14-132

**Published:** 2014-04-07

**Authors:** Sarah L Desmarais, Ashley Pritchard, Evan M Lowder, Patricia A Janssen

**Affiliations:** 1 Department of Psychology, North Carolina State University, Campus Box 7650, Raleigh NC 27695-7650, USA; 2 Department of Psychology, Simon Fraser University, 8888 University Drive Burnaby, British Columbia V5A 1S6, Canada; 3 UBC School of Population and Public Health, 2206 East Mall Vancouver, British Columbia V6T 1Z3, Canada

**Keywords:** Intimate partner abuse, Psychological aggression, Physical assault, Sexual coercion, Postpartum mental health, Stress, Anxiety, Depression, Obsessive-compulsive disorder, Posttraumatic stress disorder

## Abstract

**Background:**

Although research has established the profound effects that intimate partner abuse can have on postpartum mental health, little is known regarding how this association may change as a function of the timing and type of abuse. This study examined associations of psychological, physical and sexual abuse experienced as adults before and during pregnancy with symptoms of postpartum mental health problems in a non-clinical sample of women.

**Methods:**

English-speaking mothers aged 18 years and older in the metropolitan area of a large, Western Canadian city were recruited to participate in a study of women’s health after pregnancy. The study was advertised in hospitals, local newspapers, community venues, and relevant websites. One-hundred women completed standardized, self-report questionnaires during semi-structured interviews conducted by female research assistants at approximately 2 months postpartum. In addition to questions about their general health and well-being, participants answered questions about their experiences of intimate partner abuse and about their mental health during the postpartum period.

**Results:**

Almost two-thirds (61.0%) of women reported postpartum mental health symptoms above normal levels, with 47.0% reporting symptoms at moderate or higher levels. The majority reported some form of intimate partner abuse before pregnancy (84.0%) and more than two-thirds (70.0%), during pregnancy; however, the abuse was typically minor in nature. Multivariate models revealed that women who experienced intimate partner abuse—whether before or during pregnancy—reported higher levels of postpartum mental health problems; however, associations differed as a function of the timing and type of abuse, as well as specific mental health symptoms. Multivariate models also showed that as the number of types of intimate partner abuse experienced increased, so did the negative effects on postpartum mental health.

**Conclusions:**

Results of this study provide further evidence that intimate partner abuse is a risk factor for postpartum mental health problems. They also underscore the complex risks and needs associated with intimate partner abuse among postpartum women and support the use of integrated approaches to treating postpartum mental health problems. Future efforts should focus on the extent to which strategies designed to reduce intimate partner abuse also improve postpartum mental health and vice versus.

## Background

Postpartum mental health problems are major public health issues with significant implications for maternal and child health [1]. Indeed, this is the period during which women demonstrate the highest incidence of common mental disorders [2-4]. The most prevalent postpartum mental health problem is postpartum depression, affecting between 10% and 15% of new mothers [5-7]. However, women also are at heightened risk postpartum for other mental health problems, such as generalized anxiety disorder, obsessive-compulsive disorder and posttraumatic stress disorder, as well as psychosis [8-13]. Research has documented the profound effects untreated postpartum mental health problems can have on families and children [14]. Postpartum depression, for example, has been associated with early breastfeeding discontinuation [15], and untreated mental health problems have been associated with bonding impairment and fewer positive parenting behaviors [16]. Moreover, impaired interactions between mother(s) and children have been associated with long-term impairments in children’s cognitive and emotional development [14]. They also increase risk for maternal engagement in other risky behaviors, such as substance abuse and self-harm [17,18]. As a result, there is a need for improved knowledge and identification of risk factors for postpartum mental health problems, such as intimate partner abuse [19].

Intimate partner abuse includes “any behaviour within a current or past intimate relationship that involves (actual, attempted, or threatened) harm… that may impact or detract from the victim’s physical, psychological, sexual, economic, or spiritual well-being” [20]. Across studies, prevalence of intimate partner abuse during pregnancy has ranged anywhere from 0.9% to 20.1% [21], with variability attributable to differences in study designs, such as inclusion criteria, sample characteristics, response rates, modes of inquiry, assessment tools, and operational definitions of intimate partner abuse [22,23]. Recent population-based studies have estimated the prevalence of physical and sexual violence during pregnancy to be between 8.5% and 10.9% [22,24,25]. Overall, studies converge on the finding that risk for physical forms of intimate partner abuse is lower during pregnancy compared to other periods in women’s lives [26,27]. However, some research suggests relative risk during pregnancy compared to other timeframes may differ by type of intimate partner abuse; for example, one study of 914 pregnant women treated in health clinics in Mexico found increases in rates of emotional abuse during pregnancy, but decreases in rates of physical and sexual abuse [28].

Importantly, the postpartum period may be a particularly vulnerable time for experiencing harms associated with intimate partner abuse, with many prior studies emphasizing the physical effects of intimate partner abuse on maternal and child health [29-31]. A small but growing body of research describes postpartum mental health risks associated with intimate partner abuse [32-35]. For example, a recent study found that women exposed to intimate partner abuse during pregnancy (including physical, sexual, emotional, financial and neglect) reported rates of depression, anxiety, and stress that were approximately twice the rates reported by mothers who did not experience intimate partner abuse [36]. Another study reported that mothers of infants under 14 months reporting intimate partner abuse (defined as threat of physical harm, physical assault or forced sex) in the past 12 months (which may or may not have included pregnancy) were more likely to be diagnosed with depressive and panic disorders [9]. Examined much less frequently, psychological abuse in an intimate relationship also has been tied to depression during pregnancy [37]. Few studies, however, have reported effects of psychological aggression disaggregated from the effects of other forms of intimate partner abuse [32]. Importantly, *fear* associated with intimate partner abuse during pregnancy—independent of the abuse itself—has physical (e.g., urinary incontinence) and psychological (e.g., anxiety) consequences [38].

Despite advances in our understanding of the postpartum mental health risks associated with intimate partner abuse, extant findings are limited in several ways. For instance, the associations of psychological, physical and sexual intimate partner abuse with diverse postpartum mental health problems have been examined infrequently. When examined, studies have focused on one postpartum mental health problem, most commonly postpartum depression, to the exclusion of others [32,39]. When associations between intimate partner abuse and diverse postpartum mental health symptoms have been examined, researchers have focused on one type of abuse, typically physical assault [27], or have combined findings across types of intimate partner abuse [9]. In fact, a recent review found that more than two-thirds of existing studies report on the effects of physical violence on postpartum mental health, with fewer than half of the extant studies reporting effects by type of abuse [32]. In these studies, the definition of “postpartum” also has varied considerably within and between studies, ranging anywhere from one to 12 months or more after childbirth [30,32,36,9]; research, however, suggests the first three months postpartum is the highest risk period for the development of mental health problems [8,40,41].

Additionally, few studies have compared the effects of intimate partner abuse experienced during different periods of women’s lives—and before and during pregnancy, specifically—on postpartum mental health outcomes in the same sample of women [33,34]. This point should not be overlooked as intimate partner abuse may be differentially associated with postpartum mental health problems as a function of the timing of abuse. To demonstrate, findings of a recent meta-analysis suggest that higher rates of postpartum depression may be associated with abuse experienced during compared to before pregnancy [32]. When different time frames of abuse have been considered, study samples often comprised women already identified as being at-risk, such as urban, low income women [39] or first time, low income mothers [27]; as such, generalizability to other populations is unknown. Results of this meta-analysis also suggested differences in associations between intimate partner abuse and specific postpartum mental health problems: rates of postpartum depression appear to be higher than rates of anxiety among women who experienced intimate partner abuse at some time in their lives, though direct comparisons were not possible.

Importantly, an increased understanding of how associations between intimate partner abuse and postpartum mental health may differ as a function of the timing of abuse, type of abuse, and specific symptoms can be used to inform targeted intervention strategies. Thus, there is a need for research examining the differential effects of intimate partner abuse experienced before and during pregnancy on postpartum mental health outcomes in a non-clinical sample.

### The present study

To address these knowledge gaps, we explored the relationship between intimate partner abuse on postpartum mental health problems in a non-clinical, community-based sample of new mothers using a cross-sectional, retrospective design. Specifically, we examined the effects of psychological, physical and sexual abuse experienced as adults before and during pregnancy on symptoms of stress, anxiety, depression, obsessive compulsive disorder and posttraumatic stress disorder within the first three months postpartum. Our specific research questions were:

1. Do women who have experienced intimate partner abuse as adults before or during pregnancy have higher levels of postpartum mental health symptoms than women who have not experienced intimate partner abuse during these time frames?

2. Do associations between intimate partner abuse and postpartum mental health symptoms differ as a function of the timing of abuse (i.e., before or during pregnancy), type of abuse (i.e., psychological, physical, or sexual), or specific mental health problem (stress, anxiety, depression, obsessive compulsive disorder, and posttraumatic stress disorder)?

3. Are there cumulative effects of intimate partner abuse on postpartum mental health symptoms, such that severity of postpartum mental health symptoms increases as women experience more types of abuse?

## Methods

The Research Ethics Boards of the University of British Columbia, the Children’s and Women’s Health Centre of British Columbia, and the Vancouver Coast Health Research Institute approved this study. All participants provided written informed consent.

### Study population and recruitment

The study population was English-speaking mothers aged 18 years and older in the metropolitan area of a large, western Canadian city who were within three months postpartum. Women were recruited to participate in a one-time interview of factors that may affect women’s health and well-being after pregnancy. The study was advertised in area hospitals, local newspapers, and community venues frequented by new mothers (e.g., recreational centers, yoga and fitness studios, community centers, women’s centers), as well as on websites targeting new mothers and on websites of other relevant organizations (e.g., community websites). The recruitment materials did not specify a focus on intimate partner abuse or mental health, but rather described a broad interest in experiences occurring before and during pregnancy that may be associated with health and well-being after pregnancy. In total, 100 women participated in the study.

### Measures

#### Depression, Anxiety and Stress Scales (DASS-21)

The DASS-21 [42] is a 21-item self-report questionnaire designed to measure the severity of a range of symptom common to depression, anxiety, and tension/stress. Each of the three DASS-21 scales contains seven items. Respondents use a 4-point scale to rate the extent to which they have experienced each state over the past week. Each item is scored from 0 (did not apply to me at all) to 3 (applied to me very much or most of the time). Scores for Depression, Anxiety and Stress are calculated by summing relevant item ratings and multiplying by a factor of two. Total scores are calculated in the same way, using all items. In the present study, reliability of the scale scores ranged from adequate (α = .67 for Anxiety) to excellent (α = .87 for Stress). Reliability of the total scores was excellent (α = .89).

Severity of symptomatology was calculated for each subscale following the guidelines provided by the DASS-21 developers [42]. Specifically, for the Depression scale, scores from 0–9 indicate normal levels of depressive symptoms; scores of 10–13 indicate mild symptoms; scores of 14–20 indicate moderate symptoms; scores of 21–27 indicate severe symptoms; and scores greater or equal to 28 indicate extreme symptoms. For the Anxiety scale, scores from 0–7 indicate normal levels of depressive symptoms; scores of 8–9 indicate mild symptoms; scores of 10–14 indicate moderate symptoms; scores of 15–19 indicate severe symptoms; and scores greater or equal to 20 indicate extreme symptoms. For the Stress scale, scores from 0–7 indicate normal levels of depressive symptoms; scores of 8–9 indicate mild symptoms; scores of 10–14 indicate moderate symptoms; scores of 15–19 indicate severe symptoms; and scores greater or equal to 20 indicate extreme symptoms.

#### Yale-Brown Obsessive-Compulsive Scale (YBOCS)

The YBOCS [43,44] measures the presence and severity of obsessive-compulsive disorder symptoms. The instrument is divided into the Obsessions scale and the Compulsions scale. For each scale, five aspects of obsessive and compulsive pathology are each rated on a scale ranging from 0 (no symptoms) to 4 (extreme symptoms): time spent, degree of interference, distress, resistance (greater resistance is assigned lower scores), and perceived control over the symptom. Subscale scores are summed to yield a total score. In the present study, reliability of the Obsessions and Compulsions scales was excellent (both α = .90), as was reliability of the total scores (α = .93).

As for the DASS-21, severity of obsessive-compulsive disorder symptomatology can be calculated using the YBOCS total scores following the guidelines provided by the instrument developers [43,44]. Specifically, scores from 0–7 indicate subclinical levels of obsessive-compulsive disorder symptoms; scores of 8–15 indicate mild symptoms; scores of 16–23 indicate moderate symptoms; scores of 24–31 indicate severe symptoms; and scores greater or equal to 32 indicate extreme symptoms.

#### Posttraumatic Stress Disorder Symptom Scale (PSS-SR)

The PSS-SR [45] is a 17-item self-report measuring posttraumatic stress disorder symptomatology which directly corresponds to Diagnostic and Statistical Manual of Mental Disorders-III-Revised (DSM-III-R) criteria. For all items, symptom frequency over the preceding two weeks is reported on a 4-point scale, where 0 = not at all, 1 = once per week, 2 = 2 to 4 times per week, and 3 = 5 or more times per week. A total score is obtained by summing each symptom rating [45]. Typically, total scores of 14 or greater indicate symptom severity consistent with posttraumatic stress disorder [46,47]. Scale scores are calculated by summing symptoms in the re-experiencing (4 items), avoidance (7 items), and arousal (6 items) clusters. In the present study, reliability of the scale scores was good (α = .76-.80) and reliability of the total scores was excellent (α = .90).

#### Conflict Tactics Scale Revised (CTS-2)

The CTS2 [48] is a self-report, behavioral measure of psychological aggression (8 items), physical assault (12 items), sexual coercion (7 items), physical injury (6 items), and negotiation (6 items) occurring in the context of conflict between intimate partners. The 39 items are rated on a 8-point frequency scale (never, once, twice, 3–5 times, 6–10 times, 11–20 times, and more than 20 times, not in the past year but it did happen before) in terms of the respondent and her partner’s behaviors. This study used the CTS2 items that assessed the partner’s violent behaviors toward our participants. Because of our focus on intimate partner abuse rather than relationship behaviors more broadly, the negotiation scale items were excluded from analyses. Response options were modified to query whether or not the behavior occurred within each of two specified periods: as an adult before the woman became pregnant and during the pregnancy. Specifically, for each item, first participants were asked whether the behavior had occurred during pregnancy (yes, no), and second they were asked whether it had occurred at any point in her life prior to pregnancy (yes, no).

For each of the CTS2 scales used in this study, women were classified as having been victims of the particular type of abuse if they reported experiencing one or more of the scale items within the relevant time period. For example, a woman who reported having experienced one or more of the 8 psychological aggression items at some point prior to getting pregnant would be classified as having been a victim of psychological aggression before pregnancy. We then calculated overall prevalence (yes, no) and prevalence by type (psychological, physical, sexual) and severity (minor, severe) of intimate partner abuse for each time period following the guidelines detailed in the CTS2 coding manual [48]. We additionally created variables describing the number of types of intimate partner abuse experienced during each reference period, collapsing across participants who experienced 2 or 3 types of abuse due to small cell sizes (0, 1, 2 or 3). Reliability of the modified CTS2 scores was good to excellent (α = .75 or greater).

#### Procedures

Potential participants contacted us via secure phone or email, at which time they were screened for eligibility and interviews were scheduled. Participants completed the study measures during semi-structured, calendar-based interviews conducted by female research assistants at safe locations of the women’s choice. Briefly, calendar-based interviews are the state-of-the-art in surveying and interviewing [49-51]. These methods require respondents to retrospectively report, using a calendar as a memory aid. Personally significant and easy-to-remember landmark dates are marked on the calendar to serve as temporal anchors [50]. In the present study, the delivery date was used to calculate the approximate date of conception. These two dates then served as the primary temporal anchors. Additional anchors included dates of admission and discharge to the hospital.

Following the interviews, information regarding local mental health services and abuse resources (e.g., helpline phone numbers, shelter information, legal services, etc.) was provided as necessary. Women received an honorarium of $20 for their participation, and up to $5 for expenses associated with participation (e.g., parking, bus or cab fare).

#### Statistical analyses

We calculated descriptive statistics for all variables and conducted bivariate analyses to examine women’s mean levels of postpartum mental health symptoms by their sociodemographic characteristics (participant age, race/ethnicity, education, income, relationship status, and prior children) and by prevalence (yes, no) of each type of intimate partner abuse (psychological, physical, and sexual) both before pregnancy and during pregnancy. To answer our research questions, we then tested a series of multivariate analysis of covariance models. Specifically, to answer our first two research questions, we tested two multivariate analysis of covariance models with psychological, physical and sexual abuse (yes, no) as the dichotomous independent variables and symptoms of stress, anxiety, depression, obsessive-compulsive disorder, and posttraumatic stress disorder as the continuous dependent variables, controlling for participant characteristics. To answer our third research question, we tested two additional multivariate analysis of covariance models with number of types of intimate partner abuse experienced before and during pregnancy as the categorical predictors and symptoms of stress, anxiety, depression, obsessive-compulsive disorder, and posttraumatic stress disorder as the continuous dependent variables, again controlling for participant characteristics. Statistical significance was set at α = .05. All analyses were conducted with IBM SPSS Statistics 21.

## Results

### Descriptive statistics

Mean participant age was 32.47 years (*SD* = 4.97, range = 20-49) and that of their babies was 2.01 months (*SD* = 1.32, range = 0-5)^a^. Almost half (41.0%) already had children (biological or otherwise). The majority of respondents (71.0%) and their partners (68.4%) were Caucasian; a substantial minority were Asian (13.0% and 11.2%, respectively), which is representative of the study area. Most (96.0%) had completed high school or equivalent, and were employed (82.0%), either part-time (17.0%) or full time (65.0%). Almost two-thirds reported familial incomes of $60,000 or more (62.0%). All but five women (95.0%) were in a serious or committed relationship with the father of their new baby at the time of the interview.

Overall, almost two-thirds of women (61.0%) reported postpartum mental health symptoms that were above normal levels, with 47.0% reporting symptoms at moderate or higher levels. For stress, anxiety, depression, and obsessive-compulsive disorder, scores were above the normal range for 41.0%, 27.0%, 26.0%, and 32.0% of participants and in the clinical range for 30.0%, 20.0%, 14.0%, and 13.0% of participants, respectively. Almost one-quarter of women (22.0%) met diagnostic criteria for posttraumatic stress disorder.

The vast majority of participants reported experiencing some form of intimate partner abuse before their pregnancy (84.0%) and more than two-thirds (70.0%) reported experiencing intimate partner abuse during their pregnancy. Both before and during pregnancy, psychological aggression was most common (83.0% and 68.0%), followed by physical assault (20.0% and 12.0%) and then sexual coercion (15.0% and 12.0%). Minor abuse was more common than severe abuse in both reference periods; still, almost one-third (31.0%) of women reported severe psychological, physical and/or abuse before pregnancy, and 12.0% during pregnancy.

### Bivariate analyses

Table 1 shows postpartum mental health symptoms as a function of participant characteristics and prevalence of intimate partner abuse before and during pregnancy. Bivariate results revealed some differences in postpartum mental health symptoms as a function of participant characteristics. Significantly higher levels of anxiety and posttraumatic stress disorder symptoms were seen in women who had not completed their high school education compared to those who had completed at least high school. Significantly higher levels of anxiety and posttraumatic stress disorder symptoms also were seen in women whose annual household incomes were $60,000 or greater compared to those whose annual household incomes were less than $60,000. Women who were single showed significantly higher levels of stress, anxiety, depression, and posttraumatic stress disorder than women in relationships at the time of the interview. No significant differences in postpartum mental health symptoms were found as a function of age, race/ethnicity, or prior children; thus, these 3 participant characteristics will not be included as covariates in the multivariate analyses.

**Table 1:** Bivariate comparisons of postpartum mental health symptoms by participant characteristics

** Participant characteristics**	**Postpartum mental health symptoms**														
	**Stress**			**Anxiety**			**Depression**			**OCD**		**PTSD**			
	***M (SD)***	***95% CI***	***t***	***M (SD)***	***95% CI***	***t***	***M (SD)***	***95% CI***	***t***	***M (SD)***	***95% CI***	***t***	***M (SD)***	***95% CI***	***t***
**Age**															
Less than 33 years (*n* = 50)	14.76 (9.78)	11.98-17.54	0.43	5.72 (6.50)	3.87-7.57	1.29	5.88 (5.71)	4.26-7.50	0.24	5.90 (7.61)	3.74-8.06	0.46	7.58 (8.86)	5.06-10.10	0.27
33 years or older (*n* = 50)	13.96 (8.68)	11.49-16.43		4.32 (4.03)	3.17-5.46		5.60 (5.74)	3.97-7.23		5.26 (6.12)	3.52-7.00		7.16 (6.71)	5.25-9.07	
**Race**															
Caucasian (*n* = 71)	14.85 (9.44)	12.61-17.08	0.82	5.04 (5.52)	3.74-6.35	0.06	5.77 (5.89)	4.38-7.17	0.09	6.42 (7.43)	4.66-8.18	1.94	6.93 (7.28)	5.21-8.65	0.88
Other (*n* = 29)	13.17 (8.66)	9.88-16.47		4.97 (5.28)	2.96-6.97		5.66 (5.29)	3.64-7.67		3.52 (4.81)	1.69-5.35		8.45 (9.06)	5.00-11.86	
**Education**															
Less than high school (*n* = 4)	17.50 (12.79)	2.86-37.86	0.69	13.50 (9.57)	1.73-28.73	3.35***	11.00 (6.63)	0.44-21.55	1.91	10.00 (10.10)	6.07-26.07	1.32	19.00 (14.90)	4.71-42.71	3.17**
High school or greater (*n* = 96)	14.23 (9.10)	12.39-16.07		4.67 (4.96)	3.66-5.67		5.52 (5.59)	4.39-6.65		5.40 (6.72)	4.03-6.76		6.89 (7.13)	5.44-8.33	
**Annual Income**															
Less than $60,000 (*n* = 21)	15.2 4 (8.40)	11.41-19.06	0.18	8.29 (7.05)	5.08-11.50	3.34***	7.14 (5.78)	4.51-9.77	1.05	6.29 (8.29)	2.51-10.06	0.57	11.43 (9.76)	6.99-15.87	2.92**
$60,000 or more (*n* = 62)	14.81 (9.68)	12.35-17.26		3.87 (4.49)	2.73-5.01		5.61 (5.77)	4.15-7.08		5.31 (6.32)	3.70-6.91		5.89 (6.63)	4.20-7.57	
**Relationship Status**															
Single (*n* = 5)	22.40 (8.53)	11.81-32.99	2.03*	12.40 (6.07)	4.87-19.93	3.27***	12.40 (3.29)	8.32-16.48	2.77**	7.20 (7.33)	1.90-16.30	0.54	22.40 (8.44)	11.92-32.88	4.89***
In a relationship (*n* = 95)	13.94 (9.09)	12.09-15.79		4.63 (5.14)	3.58-5.68		5.39 (5.59)	4.25-6.53		5.49 (6.88)	4.09-6.90		6.58 (6.98)	5.16-8.00	
**Prior Children**															
No (*n* = 59)	13.46 (9.33)	11.03-15.89	1.18	4.68 (4.91)	3.40-5.96	0.75	5.22 (5.45)	3.80-6.64	1.09	6.05 (7.48)	4.10-8.00	0.82	6.59 (7.29)	4.69-8.49	1.19
Yes (*n* = 41)	15.66 (8.99)	12.82-18.50		5.51 (6.13)	3.58-7.45		6.49 (6.03)	4.58-8.39		4.90 (5.94)	3.03-6.78		8.49 (8.50)	5.80-11.17	

Note. *N* = 100. *OCD* = obsessive-compulsive disorder. *PTSD* = posttraumatic stress disorder. Comparisons are *t*-tests of the mean scores on the postpartum mental health scales. *p < .05. **p < .01. ***p < .001.

Bivariate comparisons also revealed that women who experienced intimate partner abuse—whether before or during pregnancy—reported significantly more postpartum mental health symptoms compared to those who did not experience intimate partner abuse. Psychological aggression before and during pregnancy was associated with postpartum stress. Psychological aggression during, but not before, pregnancy also was associated with symptoms of posttraumatic stress disorder. Physical assault and sexual coercion before pregnancy were associated with symptoms of anxiety, obsessive-compulsive disorder and posttraumatic stress disorder. Physical assault before pregnancy additionally was associated with symptoms of depression. Physical assault and sexual coercion during pregnancy both were associated symptoms of obsessive-compulsive disorder; however, physical assault during pregnancy also was associated with posttraumatic stress disorder, whereas sexual coercion during pregnancy was associated with anxiety. Even when significant differences in postpartum mental health symptoms as a function of intimate partner abuse were not observed, there was nonetheless a trend for increased symptomatology among women who reported abuse. Finally, as the number of types of abuse reported before and during pregnancy increased, so did symptoms of obsessive-compulsive disorder and posttraumatic stress disorder. Number of types of abuse reported before, but not during, pregnancy additionally was associated with increased symptoms of anxiety.

### Multivariate analyses

Table 2 shows results of the multivariate analysis of covariance examining the effects of psychological, physical and sexual abuse before and during pregnancy (in separate models) on symptoms of stress, anxiety, depression, obsessive-compulsive disorder, and posttraumatic stress disorder, with education, annual income, and relationship status as covariates. Only the multivariate model of physical assault during pregnancy was significant (see Table 3). However, the corrected univariate models for both intimate partner abuse before and during pregnancy were significant for stress (*F*[6, 75] = 3.87, *p* < .05, *η*_*p*_^*2*^ = .18; and *F*[6, 75] = 2.99, *p* < .01, *η*_*p*_^*2*^ = .19), anxiety (*F*[6, 75] = 2.65, *p* < .01, *η*_*p*_^*2*^ = .24; and *F*[6, 75] = 3.71, *p* < .01, *η*_*p*_^*2*^ = .23), obsessive-compulsive disorder (*F*[6, 75] = 2.42, *p* < .05, *η*_*p*_^*2*^ = .15; and *F*[6, 75] = 3.77, *p* < .01, *η*_*p*_^*2*^ = .23), and posttraumatic stress disorder (*F*[6, 75] = 5.69, *p* < .001, *η*_*p*_^*2*^ = .31; and *F*[6, 75] = 8.29, *p* < .001, *η*_*p*_^*2*^ = .30). The corrected univariate model for intimate partner abuse during, but not before, pregnancy additionally was significant for depression (*F*[6, 75] = 2.60, *p* < .05, *η*_*p*_^*2*^ = .17).

**Table 2:** Bivariate comparisons of postpartum mental health symptoms by intimate partner abuse

**Intimate partner abuse**	**Postpartum mental health symptoms**														
	**Stress**			**Anxiety**			**Depression**			**OCD**			**PTSD**		
	***M (SD)***	***95% CI***	***t***	***M (SD)***	***95% CI***	***t***	***M (SD)***	***95% CI***	***t***	***M (SD)***	***95% CI***	***t***	***M (SD)***	***95% CI***	***t***
**Before pregnancy**															
Psychological aggression															
No (*n* = 17)	10.00 (8.00)	5.89-14.11	2.18*	3.29 (4.24)	1.11-5.47	1.45	4.12 (4.61)	1.75-6.49	1.29	3.12 (5.59)	0.24-5.99	1.63	4.29 (6.23)	1.09-7.50	1.80
Yes (*n* = 83)	15.25 (9.23)	13.24-17.27		5.37 (5.60)	4.15-6.60		6.07 (5.86)	4.79-7.35		6.08 (7.04)	4.55-7.62		8.00 (8.00)	6.25-9.75	
Physical assault															
No (*n* = 80)	13.50 (9.01)	11.50-15.50	1.89	4.47 (5.00)	3.36-5.59	2.04*	5.12 (5.32)	3.94-6.31	2.20*	4.75 (6.24)	3.36-6.14	2.47*	6.04 (6.80)	4.52-7.55	3.61***
Yes (*n* = 20)	17.80 (9.42)	13.39-22.21		7.20 (6.57)	4.13-10.27		8.20 (6.58)	5.12-11.28		8.90 (8.38)	4.98-12.82		12.70 (9.44)	8.28-17.12	
Sexual coercion															
No (*n* = 85)	13.74 (9.08)	11.78-15.70	1.61	4.35 (4.68)	3.34-5.36	3.05**	5.44 (5.68)	4.21-6.66	1.28	4.68 (5.92)	3.41-5.96	3.25**	6.55 (7.43)	4.95-8.16	2.55*
Yes (*n* = 15)	17.87 (9.46)	12.63-23.10		8.80 (7.66)	4.56-13.04		7.47 (5.68)	4.32-10.61		10.67 (9.58)	5.36-15.97		12.00 (8.60)	7.24-16.76	
Number of types															
0 (*n* = 16)	10.12 (8.25)	5.73-14.52	4.21*	3.50 (4.29)	1.21-5.79	1.05	4.00 (4.73)	1.48-6.52	1.16	3.31 (5.71)	0.27-6.36	5.63**	3.87 (6.18)	0.58-7.17	3.41*
1 (*n* = 58)	13.97 (8.60)	11.71-16.23		4.38 (4.72)	3.14-5.62		5.38 (5.43)	3.95-6.81		4.67 (5.64)	3.19-6.16		6.50 (6.69)	4.74-8.26	
2 or 3 (*n* = 26)	17.85 (10.10)	13.77-21.93		7.38 (6.83)	4.63-10.14		7.62 (6.48)	5.00-10.23		9.00 (8.82)	5.44-12.56		11.46 (9.51)	7.62-15.30	
**During pregnancy**															
Psychological aggression															
No (*n* = 32)	11.31 (7.98)	8.44-14.19	2.32*	4.50 (4.74)	2.79-16.19	0.66	5.06 (4.60)	3.40-6.72	0.81	3.44 (5.45)	1.47-5.40	2.18*	5.69 (7.78)	2.88-8.49	1.48
Yes (*n* = 68)	15.79 (9.45)	13.51-18.08		5.26 (5.74)	3.88-6.21		6.06 (6.15)	4.57-7.55		6.59 (7.27)	4.83-8.35		8.16 (7.77)	6.28-10.04	
Physical assault															
No (*n* = 88)	13.82 (8.99)	11.91-15.72	1.61	4.80 (5.20)	3.69-5.90	1.12	5.39 (5.37)	4.25-6.52	1.70	4.81 (6.23)	3.49-6.13	3.18**	6.65 (7.65)	5.03-8.27	2.57*
Yes (*n* = 12)	18.33 (10.19)	11.86-24.81		6.67 (6.89)	2.29-11.05		8.33 (7.48)	3.58-13.08		11.25 (8.85)	5.62-16.88		12.67 (7.27)	8.05-17.28	
Sexual coercion															
No (*n* = 88)	13.75 (9.07)	11.83-15.67	1.81	4.55 (4.83)	3.52-5.57	2.42*	5.55 (5.62)	4.35-6.74	0.92	4.70 (5.98)	3.44-5.97	3.66***	6.84 (7.75)	5.20-8.48	1.85
Yes (*n* = 12)	18.83 (9.40)	12.86-24.80		8.50 (8.10)	3.36-13.64		7.17 (6.29)	3.17-11.17		12.00 (9.57)	5.92-18.08		11.25 (7.53)	6.46-16.04	
	***M (SD)***	***95% CI***	***F***	***M (SD)***	***95% CI***	***F***	***M (SD)***	***95% CI***	***F***	***M (SD)***	***95% CI***	***F***	***M (SD)***	***95% CI***	***F***
Number of types															
0 (*n* = 30)	11.60 (8.16)	8.55-14.65	5.94**	4.80 (4.74)	3.03-6.57	1.68	5.20 (4.66)	3.46-6.94	2.59	3.67 (5.56)	1.59-5.74	11.46***	5.70 (7.91)	2.74-8.66	7.91***
1 (*n* = 53)	14.19 (8.84)	11.75-16.63		4.34 (5.02)	2.96-5.72		5.25 (5.66)	3.69-6.81		4.58 (5.67)	3.02-6.15		6.87 (7.47)	4.81-8.93	
2 or 3 (*n* = 17)	19.76 (10.22)	14.51-25.02		7.53 (7.16)	3.85-11.21		8.24 (7.03)	4.62-11.85		12.06 (8.80)	7.53-16.58		11.88 (7.49)	8.03-15.73	

Note. *N* = 100. *OCD* = obsessive-compulsive disorder. *PTSD* = posttraumatic stress disorder. Comparisons are *t*-tests or *F*-tests, as appropriate, of the mean scores on the postpartum mental health scales. *p < .05. **p < .01. ***p < .001.

**Table 3:** Multivariate analyses of covariance of prevalence of intimate partner abuse predicting postpartum mental health problems

			**Univariate**^**b**^									
	**Multivariate**^**a**^		**Stress**		**Anxiety**		**Depression**		**OCD**		**PTSD**	
	***F***	**η**_**p**_^**2**^	***F***	**η**_**p**_^**2**^	***F***	**η**_**p**_^**2**^	***F***	**η**_**p**_^**2**^	***F***	**η**_**p**_^**2**^	***F***	**η**_**p**_^**2**^
**Model 1**												
**Intimate partner abuse before pregnancy**												
Psychological aggression	1.19	.08	3.67	.05	1.61	.02	0.73	.01	1.49	.02	3.27	.04
Physical assault	1.48	.09	1.03	.01	0.01	.00	3.53	.04	3.37	.04	3.00	.04
Sexual coercion	2.01	.12	2.32	.03	1.80	.02	0.01	.00	4.78*	.06	0.02	.00
**Covariates**												
Education	0.47	.03	0.05	.00	1.93	.02	0.59	.01	0.28	.00	0.15	.00
Annual income	2.25	.14	0.64	.01	2.02	.03	0.10	.00	1.19	.02	1.60	.02
Relationship status	1.65	.10	3.44	.04	1.96	.02	2.47	.03	0.41	.00	6.36*	.08
**Model 2**												
**Intimate partner abuse during pregnancy**												
Psychological aggression	2.03	.12	4.15*	.05	0.68	.01	0.56	.01	1.53	.02	7.41**	.09
Physical assault	2.86*	.17	1.55	.02	0.23	.00	5.59*	.07	7.16**	.09	7.48**	.09
Sexual coercion	2.24	.14	2.63	.03	1.65	.02	0.02	.00	7.31**	.09	0.09	.00
**Covariates**												
Education	0.49	.03	0.01	.00	1.44	.02	0.61	.01	0.10	.00	0.01	.00
Annual income	2.04	.13	0.64	.01	1.59	.02	0.53	.01	2.40	.03	0.79	.01
Relationship status	3.51**	.20	8.64**	.10	3.38	.04	7.24**	.09	1.17	.01	17.24***	.19

Note. Intimate partner abuse variables are the dichotomous predictors (yes, no). Postpartum mental health variables are the continuous outcomes. *OCD* = obsessive-compulsive disorder. *PTSD* = posttraumatic stress disorder. Multivariate *F* ratios were generated from Wilks’ Lambda’s statistic. ^a^Multivariate *df* = 5, 71. ^b^Univariate *df* = 1, 75. η_p_^2^ = partial eta-squared. *p < .05. **p < .01. ***p < .001.

Table 3 shows results of the multivariate analysis of covariance models examining associations between psychological, physical and sexual abuse (yes, no) before and during pregnancy (in separate models) and symptoms of stress, anxiety, depression, obsessive-compulsive disorder, and posttraumatic stress disorder as the continuous dependent variables, with education, annual income, and relationship status as covariates. After accounting for the other types of abuse and the covariates, sexual coercion was the only type of abuse experienced before pregnancy that was associated with postpartum mental health symptoms; obsessive-compulsive disorder, specifically (see Table 3). In contrast, all types of intimate partner abuse experienced during pregnancy were association with postpartum mental health symptoms. Psychological aggression during pregnancy showed independent effects on stress and posttraumatic stress disorder; physical assault during pregnancy showed independent effects on symptoms of depression, obsessive-compulsive disorder and posttraumatic stress disorder; and sexual coercion during pregnancy showed independent effects on symptoms of stress, depression, and posttraumatic stress disorder (see Table 3).

Table 4 shows results of the multivariate analysis of covariance models examining associations between the number of types of intimate partner abuse before and during pregnancy (in separate models) and symptoms of stress, anxiety, depression, obsessive-compulsive disorder, and posttraumatic stress disorder as the continuous dependent variables, with education, annual income, and relationship status as covariates. The multivariate model for abuse during, but not before pregnancy, was significant (see Table 4). In terms of the specific postpartum mental health problems, univariate models indicated that symptoms of stress, obsessive-compulsive disorder and posttraumatic stress disorder increased with the number of types of abuse experienced, both before and during pregnancy (see Table 4).

**Table 4:** Multivariate analyses of covariance of number of types of intimate partner abuse predicting postpartum mental health problems

			**Univariate**^**b**^									
	**Multivariate**^**a**^		**Stress**		**Anxiety**		**Depression**		**OCD**		**PTSD**	
	***F***	**η**_**p**_^**2**^	***F***	**η**_**p**_^**2**^	***F***	**η**_**p**_^**2**^	***F***	**η**_**p**_^**2**^	***F***	**η**_**p**_^**2**^	***F***	**η**_**p**_^**2**^
**Model 1**												
**Intimate partner abuse before pregnancy**												
Number of types	1.76	.11	4.21*	.10	1.05	.03	1.16	.03	5.63**	.13	3.41*	.08
**Covariates**												
Education	0.48	.03	0.03	.00	1.73	.02	0.68	.01	0.16	.00	0.20	.00
Annual income	2.40*	.14	0.18	.00	3.43	.04	0.00	.00	0.13	.00	2.64	.03
Relationship status	1.96	.12	2.84	.04	1.40	.02	3.16	.04	0.90	.01	7.25**	.09
**Model 2**												
**Intimate partner abuse during pregnancy**												
Number of types	3.51***	.20	5.94**	.13	1.68	.04	2.59	.06	11.46***	.23	7.91**	.17
**Covariates**												
Education	0.53	.03	0.00	.00	1.81	.02	0.65	.01	0.16	.00	0.04	.00
Income	2.27	.14	0.32	.00	2.69	.03	0.09	.00	0.55	.01	2.28	.03
Relationship Status	3.24*	.18	8.19**	.10	3.08	.04	6.24*	.08	0.35	.00	15.98***	.17

Note. Intimate partner abuse variables are the categorical predictors (0, 1, 2 or 3). Postpartum mental health variables are the continuous outcomes. *OCD* = obsessive-compulsive disorder. *PTSD* = posttraumatic stress disorder. Multivariate *F* ratios were generated from Wilks’ Lambda’s statistic. ^a^Multivariate *df* = 5, 72. ^b^Univariate *df* = 1, 76. η_p_^2^ = partial eta-squared. *p < .05. **p < .01. ***p < .001.

## Discussion

The present study explored associations of psychological, physical and sexual intimate partner abuse among postpartum mental health in a non-clinical, community-based sample of new mothers using a retrospective, cross-sectional design. Though many studies have examined associations between intimate partner abuse and postpartum mental health [32], this study adds to the empirical literature by disaggregating effects of psychological, physical and sexual abuse experienced at two different times during women’s lives, namely before and during pregnancy, on diverse postpartum mental health problems.

Multivariate analyses showed important differences in the impact of intimate partner abuse on postpartum mental health as a function of the type and timing of abuse. Psychology aggression during, but not before, pregnancy was associated with symptoms of stress and posttraumatic stress disorder, whereas sexual coercion, both before and during pregnancy, was associated with symptoms of obsessive-compulsive disorder. Physical assault during pregnancy appeared to have the greatest impact on postpartum mental health and was associated with depression, obsessive-compulsive disorder, and posttraumatic stress disorder when it occurred during pregnancy. However, we found no independent effects of physical assault before pregnancy on postpartum mental health, after accounting for psychological aggression, sexual coercion, participant education, annual income, and relationship status. This latter finding is inconsistent with prior studies and may be attributable to the failure of most extant research to account for experiences of non-physical types of intimate partner abuse in their models [32].

Consistent with the findings of prior meta-analytic work, we observed stronger associations between abuse experienced during compared to before pregnancy [32], though we extended this pattern of results from depression and posttraumatic stress disorder to stress and obsessive-compulsive disorder and posttraumatic stress disorder. In contrast with prior research, associations between intimate partner abuse (any type) and symptoms of postpartum anxiety were not significant after controlling for participant education, annual income, and relationship status [32]. Multivariate analyses also showed that risk for postpartum mental health problems—specifically, stress, obsessive-compulsive disorder and posttraumatic stress disorder—increases as the number of types of intimate partner abuse increases. To our knowledge, this is the first study to demonstrate the cumulative effects of intimate partner abuse on postpartum mental health.

Our results build upon prior empirical evidence linking exposure to psychological intimate partner abuse with postpartum mental health problems [37,52]; indeed, psychological aggression during pregnancy was associated with higher rates of both stress and posttraumatic stress disorder. Given the high rates of exposure and mental health sequelae, there is need to conduct routine screening for non-physical forms of abuse during pregnancy. Moreover, results suggest that knowledge of whether women have experienced psychological, physical and/or sexual abuse in an intimate relationship may assist health care professionals to target postpartum mental health treatment and intervention efforts.

This is one of the few studies to examine associations between intimate partner abuse and postpartum mental health in a non-clinical as opposed to a hospital, clinic or at-risk sample. Our analyses have revealed high rates of intimate partner abuse and postpartum mental health problems. More than two-thirds of women in this non-clinical sample reported elevated levels of postpartum mental health symptoms, and almost half reported symptoms that were moderate in severity or higher. These rates are slightly higher but generally in keeping with those reported elsewhere [3,5,7,10,13]. In contrast, rates of intimate partner abuse, both before and during pregnancy, were notably higher than those reported in recent population-based research [22,24,25]. Inconsistencies may be attributable to measurement differences: we used a modified version of the CTS-2 to measure intimate partner abuse, whereas most prior work has relied on measures with fewer items and/or that are restricted to moderate to severe intimate partner abuse [27]. Studies employing the CTS-2 tend to find higher rates of intimate partner abuse compared to those using other measures [23]. Additionally, most studies have focused on physical intimate partner abuse, our operational definition included not only physical assault, but also psychological aggression and sexual coercion [21,32]. Indeed, examining severe physical intimate partner abuse alone, we see rates more consistent with those reported in prior research [22,27,29]. Finally, we took great care to establish rapport with participants prior to interview administration and the questions regarding postpartum mental health and intimate partner abuse were placed about two-thirds of the way through the interview; these two strategies may have increased the degree to which women were willing to disclose their experiences.

The present study is limited in several ways. First, the data are cross-sectional and do not afford longitudinal analyses of the effects of intimate partner abuse on women’s mental health over time. Future research should examine the long-term unique and cumulative impact of psychological, physical and sexual intimate partner abuse on maternal and child health over time. Second, we employed convenience sampling; generalizability of experiences reported by women who volunteered for a study on health after pregnancy is unknown. Third, our sample was relatively small and power to detect significant associations in our analyses may be limited. Fourth, we focused on whether or not women experienced intimate partner abuse, rather than frequency of intimate partner abuse over time. Fourth, our data were derived from retrospective, self-reports and may be susceptible to recall bias and errors, as well as social desirability [23]. Yet, retrospective, self-report has been shown to be a valid and reliable method for collecting sensitive data, including reports of postpartum mental health problems [53] and intimate partner abuse during the perinatal period [54]. Fifth, and finally, our data on intimate partner abuse experienced before and during pregnancy were collected at the same time. The ideal approach would have been to interview women at multiple times before and during the perinatal period; however, this was not possible. To enhance the quality of data given our study constraints, we employed a calendar-based interviewing approach, which has been shown to increase reliability and validity of retrospective, behavioral reports [49-51].

## Conclusion

Results of this study provide further evidence that intimate partner abuse is a risk factor for postpartum mental health problems. A majority of women, even in a non-clinical, community-based sample, retrospectively reported experiences of intimate partner abuse that occurred both before and during pregnancy. As such, there is a critical need for the development, implementation and evaluation of strategies designed to reduce intimate partner abuse during the perinatal period. The perinatal period presents a unique opportunity to identify women who may be at risk for both intimate partner abuse, as well as postpartum mental health problems, because it brings otherwise healthy women contact with health care providers [9]. However, in contrast to the significant body of research evaluating screening and assessment strategies, there is a dearth of empirical evidence supporting the effectiveness of intimate partner abuse intervention and prevention strategies [55]; this remains an important avenue for future research.

## Endnote

^a^Though women were recruited within 0–3 months postpartum, some interviews were completed after 3 months postpartum (*n* = 12) due to scheduling conflicts.

## Competing interests

The authors declare that they have no competing interests.

## Authors’ contributions

SLD and PAJ conceived of the study. SLD oversaw all aspects of the study design, data collection, and analysis of the data. AP participated in the study coordination and data collection. EML contributed to the manuscript revisions. All authors contributed to the preparation of the manuscript, and read and approved the final manuscript.

## Authors’ information

SLD is an Assistant Professor in the Psychology in the Public Interest Program at North Carolina State University. Her program of research focuses on issues at the nexus of psychology and law with special emphasis on issues related to mental health, violence and victimization. AP is a graduate student in the Law and Forensic Psychology Program at Simon Fraser University, with research interests in the co-occurrence of violence and victimization. ELM is a graduate student in the Psychology in the Public Interest Program at North Carolina State University, with research interests in evaluating alternatives to incarceration for justice-involved adults with serious mental illnesses. PAJ is Director of the MPH Program and Co-Leader of the Maternal Child Health Theme in the School of Population & Public Health at the University of British Columbia. Her areas of research include maternal, fetal and newborn health; women’s health; and violence.

## Pre-publication history

The pre-publication history for this paper can be accessed here:

http://www.biomedcentral.com/1471-2393/14/132/prepub

## References

[B1] Ross-Davie M Elliot S Sarkar A Green L A public health role in perinatal mental health: Are midwives ready? Br J Midwifery 2006 14 6 330 334

[B2] Fisher J Cabral de Mello M Patel V Rahman A Tran T Holton S Holmes W Prevalence and determinants of common perinatal mental disorders in women in low- and lower-middle-income countries: a systematic review Bull World Health Organ 2012 90 2 139 149 10.2471/BLT.11.091850 PMC3302553 22423165

[B3] Watson JP Elliott SA Rugg AJ Brough DI Psychiatric-Disorder in pregnancy and the 1st postnatal year Br J Psychiatry 1984 144 May 453 462 6733369 10.1192/bjp.144.5.453

[B4] Evans J Heron J Francomb H Oke S Golding O Parents ALS Cohort study of depressed mood during pregnancy and after childbirth Br Med J 2001 323 7307 257 260 10.1136/bmj.323.7307.257 11485953 PMC35345

[B5] Gavin NI Gaynes BN Lohr KN Meltzer-Brody S Gartlehner G Swinson T Perinatal depression - a systematic review of prevalence and incidence Obstet Gynecol 2005 106 5 1071 1083 10.1097/01.AOG.0000183597.31630.db 16260528

[B6] Henderson JJ Evans SF Straton JAY Priest SR Hagan R Impact of postnatal depression on breastfeeding duration Birth 2003 30 3 175 180 10.1046/j.1523-536X.2003.00242.x 12911800

[B7] O’Hara M Swain A Rates and risk of postpartum depression: a meta-analysis Int Rev 1996 8 1 37 54

[B8] Brockington I Postpartum psychiatric disorders Lancet 2004 363 9405 303 310 10.1016/S0140-6736(03)15390-1 14751705

[B9] Cerulli C Talbot NL Tang W Chaudron LH Co-Occurring intimate partner violence and mental health diagnoses in perinatal women J Womens Health 2011 20 12 1797 1803 10.1089/jwh.2010.2201 PMC3278805 21923282

[B10] Ross LE McLean LM Anxiety disorders during pregnancy and the postpartum period: A systematic review J Clin Psychiatry 2006 67 8 1285 1298 10.4088/JCP.v67n0818 16965210

[B11] Fairbrother N Abramowitz JS New parenthood as a risk factor for the development of obsessional problems Behav Res Ther 2007 45 9 2155 2163 10.1016/j.brat.2006.09.019 17084810

[B12] Wu Q Chen HL Xu XJ Violence as a risk factor for postpartum depression in mothers: a meta-analysis Arch Womens Ment Health 2012 15 2 107 114 10.1007/s00737-011-0248-9 22382278

[B13] Zambaldi CF Cantilino A Montenegro AC Paes JA de Albuquerque TLC Sougey EB Postpartum obsessive-compulsive disorder: prevalence and clinical characteristics Compr Psychiatry 2009 50 6 503 509 10.1016/j.comppsych.2008.11.014 19840587

[B14] Letourneau N Morris CY Stewart M Hughes J Critchley KA Secco L Social Support needs identified by mothers affected by intimate partner violence J Interpers Violence 2013 29 14 2873 2893 23686618 10.1177/0886260513488685

[B15] Taveras EM Capra AM Braveman PA Jensvold NG Escobar GJ Lieu TA Clinician support and psychosocial risk factors associated with breastfeeding discontinuation Pediatrics 2003 112 1 Pt 1 108 115 12837875 10.1542/peds.112.1.108

[B16] Muzik M Bocknek EL Broderick A Richardson P Rosenblum KL Thelen K Seng JS Mother-infant bonding impairment across the first 6 months postpartum: The primacy of psychopathology in women with childhood abuse and neglect histories Arch Womens Ment Health 2013 16 1 29 38 10.1007/s00737-012-0312-0 23064898 PMC4040083

[B17] Coleman-Cowger VH Mental health treatment need among pregnant and postpartum women/girls entering substance abuse treatment Psychol Addict Behav 2012 26 2 345 350 21895350 10.1037/a0025355

[B18] Lincoln A Feyerharm R Damron P DeVault M Lorenz D Dooley S Maternal depression after delivery in Oklahoma J Okla State Med Assoc 2008 101 12 307 311 19177992

[B19] Oates M Perinatal psychiatric disorders: a leading cause of maternal morbidity and mortality Br Med Bull 2003 67 219 229 10.1093/bmb/ldg011 14711766

[B20] Desmarais SL Gibas A Nicholls TL Ferguson CF Beyond violence against women: Gender-inclusiveness in domestic violence research, policy, and practice Violent crime: Clinical and social implications 2009 CA: Sage: Thousand Oaks 184 206

[B21] Taillieu T Brownridge DA Prevalence, patters, and risk factors for experiencing intimate partner violence during pregnancy: a review of the literature Aggress Violent Behav 2010 15 1 14 35 10.1016/j.avb.2009.07.013

[B22] Daoud N Urquia ML O’Campo P Heaman M Janssen PA Smylie J Thiessen K Prevalence of abuse and violence before, during, and after pregnancy in a national sample of Canadian women Am J Public Health 2012 102 10 1893 1901 10.2105/AJPH.2012.300843 22897526 PMC3490644

[B23] Desmarais SL Reeves KA Nicholls TL Telford RP Fiebert MS Prevalence of physical violence in intimate relationships: Part 1. Rates of male and female victimization Partner Abuse 2012 3 140 169 10.1891/1946-6560.3.2.140

[B24] Saltzman LE Johnson CH Gilbert BC Goodwin MM Physical abuse around the time of pregnancy: an examination of prevalence and risk factors in 16 states Matern Child Health J 2003 7 1 31 43 10.1023/A:1022589501039 12710798

[B25] Guo SF Wu JL Qu CY Yan RY Physical and sexual abuse of women before, during, and after pregnancy Int J Gynaecol Obstet 2004 84 3 281 286 10.1016/j.ijgo.2003.08.019 15001384

[B26] Martin SL Mackie L Kupper LL Buescher PA Moracco KE Physical abuse of women before, during, and after pregnancy JAMA 2001 285 12 1581 1584 10.1001/jama.285.12.1581 11268265

[B27] Scribano PV Stevens J Kaizar E Team N-IR The effects of intimate partner violence before, during, and after pregnancy in nurse visited first time mothers Matern Child Health J 2013 17 2 307 318 10.1007/s10995-012-0986-y 22426619

[B28] Castro R Peek-Asa C Ruiz A Violence against women in Mexico: a study of abuse before and during pregnancy Am J Public Health 2003 93 7 1110 1116 10.2105/AJPH.93.7.1110 12835194 PMC1447918

[B29] El Kady D Gilbert WM Xing G Smith LH Maternal and neonatal outcomes of assaults during pregnancy Obstet Gynecol 2005 105 2 357 363 10.1097/01.AOG.0000151109.46641.03 15684165

[B30] Janssen PA Holt VL Sugg NK Emanuel I Critchlow CM Henderson AD Intimate partner violence and adverse pregnancy outcomes: a population-based study Am J Obstet Gynecol 2003 188 5 1341 1347 10.1067/mob.2003.274 12748509

[B31] Lipsky S Holt VL Easterling TR Critchlow CW Police-reported intimate partner violence during pregnancy and the risk of antenatal hospitalization Matern Child Health J 2004 8 2 55 63 15198172 10.1023/b:maci.0000025727.68281.aa

[B32] Howard LM Oram S Galley H Trevillion K Feder G Domestic Violence and perinatal mental disorders: A systematic review and meta-analysis PLoS Med 2013 10 5 e1001452 10.1371/journal.pmed.1001452 23723741 PMC3665851

[B33] Janssen PA Heaman MI Urquia ML O’Campo PJ Thiessen KR Risk factors for postpartum depression among abused and nonabused women Am J Obstet Gynecol 2012 207 6 489 e481 23063016 10.1016/j.ajog.2012.09.022

[B34] Urquia ML O’Campo PJ Heaman MI Janssen PA Thiessen KR Experiences of violence before and during pregnancy and adverse pregnancy outcomes: an analysis of the Canadian Maternity Experiences Survey BMC Pregnancy Childbirth 2011 11 42 10.1186/1471-2393-11-42 21649909 PMC3141595

[B35] Beydoun HA Al-Sahab B Beydoun MA Tamim H Intimate partner violence as a risk factor for postpartum depression among Canadian women in the Maternity Experience Survey Ann Epidemiol 2010 20 8 575 583 10.1016/j.annepidem.2010.05.011 20609336 PMC4179881

[B36] Malta LA McDonald SW Hegadoren KM Weller CA Tough SC Influence of interpersonal violence on maternal anxiety, depression, stress and parenting morale in the early postpartum: a community based pregnancy cohort study BMC Pregnancy Childbirth 2012 12 153 10.1186/1471-2393-12-153 23241428 PMC3544728

[B37] Tiwari A Chan KL Fong D Leung WC Brownridge DA Lam H Wong B Lam CM Chau F Chan A Cheung KB Ho PC The impact of psychological abuse by an intimate partner on the mental health of pregnant women BJOG 2008 115 3 377 384 10.1111/j.1471-0528.2007.01593.x 18190375 PMC2253706

[B38] Brown SJ McDonald EA Krastev AH Fear of an intimate partner and women’s health in early pregnancy: Findings from the Maternal Health Study Birth 2008 35 4 293 302 10.1111/j.1523-536X.2008.00256.x 19036042

[B39] Faisal-Cury A Menezes PR D’Oliveira AF Schraiber LB Lopes CS Temporal relationship between intimate partner violence and postpartum depression in a sample of low income women Matern Child Health J 2012 17 7 1297 1303 22935913 10.1007/s10995-012-1127-3

[B40] Andersson L Sundstrom-Poromaa I Wulff M Astrom M Bixo M Depression and anxiety during pregnancy and six months postpartum: a follow-up study Acta Obstet Gynecol Scand 2006 85 8 937 944 10.1080/00016340600697652 16862471

[B41] Cooper PJ Murray L Postnatal depression BMJ 1998 316 7148 1884 1886 10.1136/bmj.316.7148.1884 9632411 PMC1113362

[B42] Lovibond PF Lovibond SH The structure of negative emotional states - Comparison of the Depression Anxiety Stress Scales (DASS) with the Beck Depression and Anxiety Inventories Behav Res Ther 1995 33 3 335 343 10.1016/0005-7967(94)00075-U 7726811

[B43] Goodman WK Price LH Rasmussen SA Mazure C Delgado P Heninger GR Charney DS The Yale-Brown Obsessive Compulsive Scale: 2. Validity Arch Gen Psychiatry 1989 46 11 1012 1016 10.1001/archpsyc.1989.01810110054008 2510699

[B44] Goodman WK Price LH Rasmussen SA Mazure C Fleischmann RL Hill CL Heninger GR Charney DS The Yale-Brown Obsessive Compulsive Scale: 1. Development, Use, and Reliability Arch Gen Psychiatry 1989 46 11 1006 1011 10.1001/archpsyc.1989.01810110048007 2684084

[B45] Foa EB Riggs DS Dancu CV Rothbaum BO Reliability and validity of a brief instrument for assessing posttraumatic-stress-disorder J Trauma Stress 1993 6 4 459 473 10.1002/jts.2490060405

[B46] Sin GL Abdin E Lee J The PSS-SR as a screening tool for PTSD in first-episode psychosis patients Early Interv Psychiatry 2012 6 2 191 194 10.1111/j.1751-7893.2011.00327.x 22240016

[B47] Coffey SF Gudmundsdottir B Beck JG Palyo SA Miller L Screening for PTSD in motor vehicle accident survivors using the PSS-SR and IES J Trauma Stress 2006 19 1 119 128 10.1002/jts.20106 16568464 PMC1424666

[B48] Straus MA Hamby SL Boney-McCoy S Sugarman DB Revised Conflict Tactics Scale: development and preliminary psychometric data J Fam Issues 1996 17 3 283 316 10.1177/019251396017003001

[B49] Belli RF The structure of autobiographical memory and the event history calendar: potential improvements in the quality of retrospective reports in surveys Memory 1998 6 4 383 406 10.1080/741942610 9829098

[B50] Belli RF Shay WL Stafford FP Event history calendars and question list surveys: a direct comparison of interviewing methods Public Opin Q 2001 65 1 45 74 10.1086/320037 11264054

[B51] Schwarz N Oyserman D Asking questions about behavior: cognition, communication, and questionnaire construction Am J Eval 2001 22 2 127 160

[B52] Ludemir AB Lewis GH Valongueiro SA de Araujo TV Araya R Violence against women by their intimate partner during pregnancy and postnatal depression: A prospective cohort study Lancet 2010 376 9744 903 910 10.1016/S0140-6736(10)60887-2 20822809

[B53] Boyd RC Le HN Somberg R Review of screening instruments for postpartum depression Arch Womens Ment Health 2005 8 3 141 153 10.1007/s00737-005-0096-6 16133785

[B54] Webster J Holt V Screening for partner violence: direct questioning or self-report? Obstet Gynecol 2004 103 2 299 303 10.1097/01.AOG.0000110245.83404.3d 14754699

[B55] Jahanfar S Janssen PA Howard LM Dowswell T Interventions for preventing or reducing domestic violence against pregnant women Cochrane Database Systematic Review 2011 10.1002/14651858.CD009414.pub3 PMC7104547 25390767

